# Effects of the application of low-temperature atmospheric plasma on titanium implants on wound healing in peri-implant connective tissue in rats

**DOI:** 10.1186/s40729-024-00524-3

**Published:** 2024-03-21

**Authors:** Atsuro Harada, Hodaka Sasaki, Yosuke Asami, Kiyotoshi Hanazawa, Sota Miyazaki, Hideshi Sekine, Yasutomo Yajima

**Affiliations:** 1 Department of Oral and Maxillofacial Implantology, Tokyo Dental College, 2-9-18 Kandamisaki-Cho, Chiyoda-Ku, Tokyo, 101-0061 Japan; 2 Oral Health Science Center, Tokyo Dental College, 2-9-18 Kandamisaki-Cho, Chiyoda-Ku, Tokyo, 101-0061 Japan; 3 Department of Fixed Prosthodontics, Tokyo Dental College, 2-9-18 Kandamisaki-Cho, Chiyoda-Ku, Tokyo, 101-0061 Japan

**Keywords:** Dental implant, Handheld low-temperature atmospheric pressure plasma, Peri-implant connective tissue, Wound healing, Collagen, Integrin

## Abstract

**Purpose:**

This study aimed to clarify the effects of surface modification of titanium (Ti) implants by low-temperature atmospheric pressure plasma treatment on wound healing and cell attachment for biological sealing in peri-implant soft tissue.

**Methods:**

Hydrophilization to a Ti disk using a handheld low-temperature atmospheric pressure plasma device was evaluated by a contact angle test and compared with an untreated group. In in vivo experiments, plasma-treated pure Ti implants using a handheld plasma device (experimental group: PL) and untreated implants (control group: Cont) were placed into the rat upper molar socket, and samples were harvested at 3, 7 and 14 days after surgery. Histological evaluation was performed to assess biological sealing, collagen- and cell adhesion-related gene expression by reverse transcription quantitative polymerase chain reaction, collagen fiber detection by Picrosirius Red staining, and immunohistochemistry for integrins.

**Results:**

In in vivo experiments, increased width of the peri-implant connective tissue (PICT) and suppression of epithelial down growth was observed in PL compared with Cont. In addition, high gene expression of types I and XII collagen at 7 days and acceleration of collagen maturation was recognized in PL. Strong immunoreaction of integrin α2, α5, and β1 was observed at the implant contact area of PICT in PL.

**Conclusions:**

The handheld low-temperature atmospheric pressure plasma device provided hydrophilicity on the Ti surface and maintained the width of the contact area of PICT to the implant surface as a result of accelerated collagen maturation and fibroblast adhesion, compared to no plasma application.

## Background

Recently, dental implants have been widely and successfully used as prosthodontic treatment for missing teeth. Accordingly, the number of implant complications is increasing. In particular, peri-implantitis caused by bacterial infection has been observed in many cases, with prevalence reported to be 9–47% [1–4]. Dental implants placed in jaw bone with penetration of oral mucosa are always at risk of infection due to the bacterial-rich environment in the oral cavity [5], which can lead to peri-implant bone resorption as peri-implantitis. In addition, biological sealing against bacterial invasion in peri-implant tissues has been reported to be more vulnerable than that in natural periodontal tissues [6]. Furthermore, it has also been reported that the complication rates involving postoperative infection in these cases were 6.5% and 77%, respectively, within 1 month after implant placement [7]. Therefore, improving the defensive mechanisms of peri-implant soft tissues against bacterial infection as a starting point has been attracting attention to establish more effective strategies for the prevention of peri-implantitis.

Peri-implant soft tissue is formed by dental implant placement with penetration of the mucosa, and serves as an important barrier against infection from the oral environment [8]. It is also considered a protective barrier between the oral environment and alveolar bone that contributes to the maintenance of osseointegration [9]. Peri-implant soft tissue consists of epithelium (peri-implant epithelium) and connective tissue (peri-implant connective tissue [PICT]), which morphologically resemble the periodontal tissue of natural teeth. However, there are some molecular biological differences in peri-implant soft tissue when compared with natural periodontal tissue. Peri-implant epithelium has been reported to express laminin-5, a cell adhesion molecule, less than the junctional epithelium of natural teeth [10], and to be less resistant to invasive foreign substances [11]. In PICT, collagen fibers run parallel to the implant [12], and the blood supply is lower than that in the connective tissue of gingiva due to the lack of a periodontal ligament [6]. In addition, peri-implant attachments with a width of 2 mm and connective tissue attachments with a width of 1–1.5 mm have been reported [12]. During the formation of peri-implant soft tissue after implant surgery, the epithelium tends to exhibit downgrowth along the implant surface, thereby providing a pathway for the invasion of external pathogens [13] and inducing peri-implant bone resorption [14–16]. Alternatively, the presence of PICT has been reported to be important for inhibiting epithelial downgrowth [12, 17]. Therefore, regulating the formation of the peri-implant epithelium and connective tissue can be a crucial factor for controlling infections in peri-implant tissue.

In recent years, to improve the defensive mechanisms of peri-implant soft tissue, many studies have focused on the surface properties of dental implant materials, such as topography, chemical properties, surface charge, and wettability. In particular, the addition of hydrophilic properties to implants/abutments by surface modification procedures seems to improve the defensive mechanisms of peri-implant by increasing cell attachment [18]. Some procedures for surface modification, including both physical modification methods (e.g., low-temperature plasma treatment [19], ultraviolet application [20]) and chemical modification methods (e.g., sandblasted, large grit, acid-etched implants stored in 0.9% NaCl solution [21], hydrogen peroxide immersion method [22], sodium hydroxide solution treatment [23]) have been reported to impart hydrophilicity to dental implant materials such as titanium (Ti). In particular, physical modification methods are expected to have clinical applications because it is relatively easy to obtain a hydrophilic surface. One physical modification method—low-temperature plasma treatment—has been applied to biomaterials in dentistry, including dental implants, for surface modification. The application of low-temperature atmospheric pressure plasma provided a hydrophilic surface on Ti by producing reactive oxygen species with high voltage to the atmosphere and removing hydrocarbons [24]. Furthermore, low-temperature atmospheric pressure plasma application has been reported to inactivate the bacteria and activate fibroblast proliferation around wounds [25, 26]. In dentistry, there have been reports of plasma application for sterilizing instruments [27], improving the bond strength of dentin [28], inhibiting periodontal pathogenic bacteria growth [29], and application in drug delivery systems [30, 31]. It has also enhanced the proliferation of osteoblast-like cells on the Ti surface, increased bone-to-implant contact in vivo, and promoted osseointegration [19]. However, the large size of plasma devices used in the past has made application to the clinical setting inconvenient. Recently, small, handheld, low-temperature atmospheric pressure plasma devices have been developed, and are expected to be applied on the chair side of clinical dental treatment, including dental implants. Handheld, low-temperature atmospheric pressure plasma treatment on Ti implant surfaces has also been shown to accelerate osseointegration, similar to larger devices [32, 33]. However, the effects of surface modification by handheld, low-temperature atmospheric pressure plasma treatment on the peri-implant soft tissue area remain unclear. Imparting hydrophilicity at the implant surface of the mucosal-penetrated area by plasma treatment could accelerate wound healing and promote cell attachment in the peri-implant soft tissue area.

Given this background, the present study aimed to clarify how surface modification of Ti implants using handheld-type low-temperature atmospheric pressure plasma treatment affects wound healing and cell attachment for biological sealing in peri-implant soft tissue.

## Methods

### Evaluation of hydrophilization on a Ti disk using a handheld-type low-temperature atmospheric pressure plasma device

**Fig. 1:**
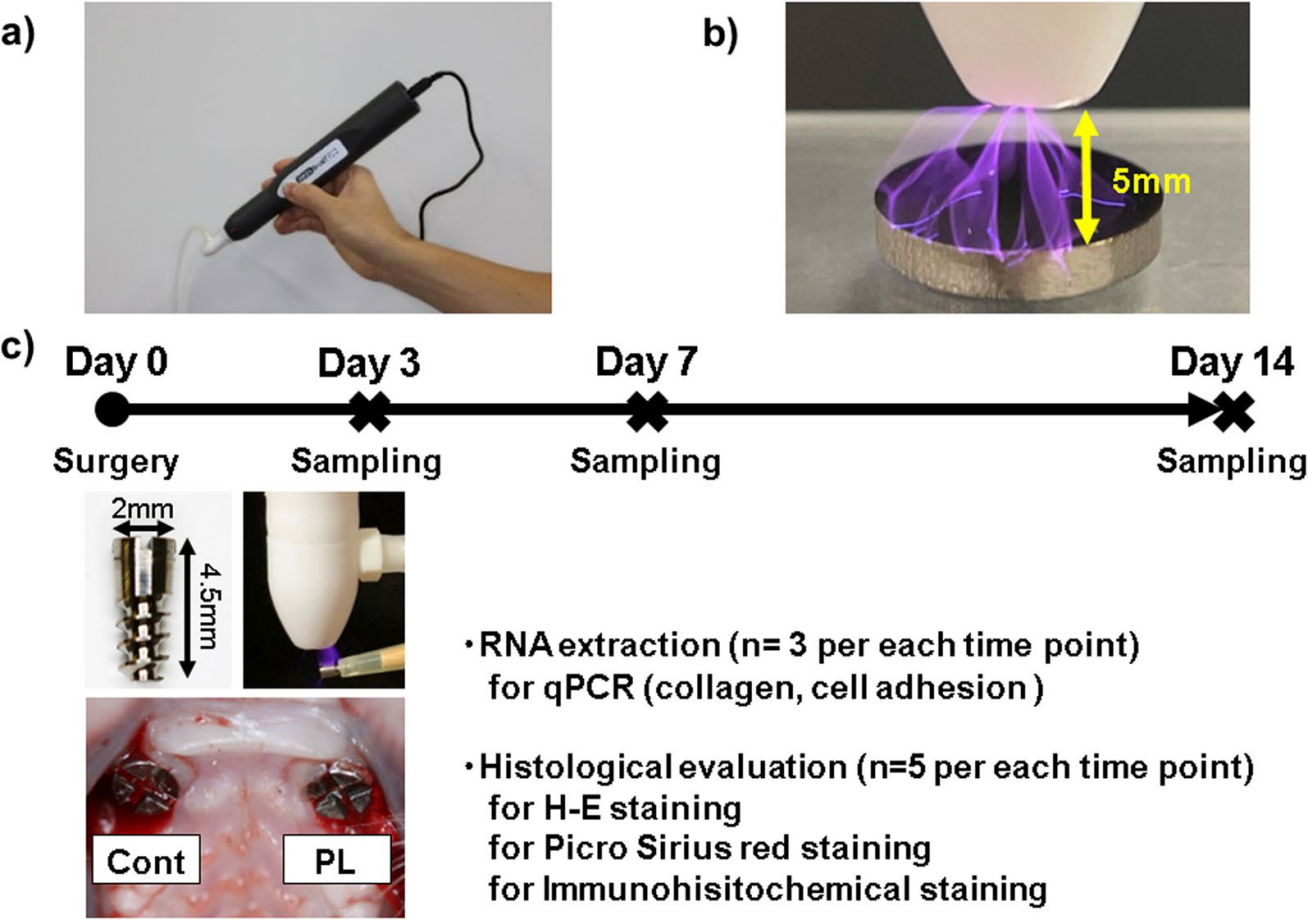
Schema of the time schedule for tissue sampling. **a** Photograph of the plasma device. **b** The distance between the plasma device and Ti disk was set to 5 mm, and plasma treatment was performed for 30 s. **c** The sampling time point is indicated as a cross. An implant (diameter 2 mm, length 4.5 mm), plasma treatment to the implant, and an intraoral photograph after implant placement are shown

A small, handheld low-temperature atmospheric pressure plasma device (Piezobrush^®^ PZ2; input power: 30 W, Relyon Plazma GmbH, Regensburg, Germany) with air was used in this study (Fig. 1a). Commercial pure titanium (CpTi) disks (JIS grade 4; Tokyo Titanium, Saitama, Japan; diameter: 13 mm, thickness 2.54 mm) were used to evaluate the effect of plasma application to Ti. The CpTi disks were polished roughly with waterproof polishing paper (#120, #240, #320, #400, #600, #800, and #1200) using a polishing machine (Ecomet 3; Buehler, Lake Bluff, IL, USA) and finished with polishing cloth (3 μm diamond particles and 0.6 μm colloidal silica). All samples were ultrasonically cleaned with acetone for 10 min, 95% ethanol for 10 min, and distilled water for 10 min before use in the experiments. Prior to evaluation, theses samples aseptically stored at room temperature for 1 week to exclude effect of plasma application. The distance between the plasma device and Ti disk was set to 5 mm, and hydrophilizing by plasma treatment was performed for 30 s (Fig. 1b). The evaluation of macro- and micro-level surface observation, wettability, roughness, and temperature change in the Ti disks after plasma treatment was performed using a handheld plasma device (PL) or no treatment (Cont). The micro-level surface of the Ti disks was observed by scanning electron microscopy (SU6600; Hitachi, Tokyo, Japan). The wettability of the Ti disk surface after plasma treatment was evaluated using a contact angle measurement. The pure water droplet volume was 1 μL and the contact angle was measured after 5 s using a contact angle meter (Phoenix α; Meiwa-forces, Tokyo, Japan; *n* = 5 per group). The two-dimensional arithmetic mean surface roughness (Ra), with a length of 645 μm (cutoff value: 250 μm), and the three-dimensional arithmetic mean roughness (Sa), with a range of 645 × 645 μm (cutoff value: 250 μm) and a cutoff value of 250 × 250 μm, were measured using a three-dimensional laser microscope (LEXT OLS4000; Olympus, Tokyo, Japan; *n* = 5 per each group). The temperature change of the Ti disk surface was measured using an infrared noncontact thermometer for 5 s after plasma treatment (THK-TOP01; Tahoco, Fujian, China; *n* = 5 per group).

### Dental implant surgery

Five-week-old male Sprague Dawley rats weighing approximately 150 g (Japan SLC Co., Ltd., Tokyo, Japan) were used in this experiment. The bilateral maxillary first molars were extracted under general anesthesia using a combination anesthetic (0.375 mg/kg medetomidine hydrochloride, Nihon Zenyaku Kogyo, Fukushima, Japan; 2.0 mg/kg midazolam, Fuji Pharmaceutical, Tokyo, Japan; and 2.5 mg/kg butorphanol tartrate, Meiji Seika, Tokyo, Japan). For Cont, the Ti implant (JIS grade 4 commercially pure titanium, 2 mm in diameter and 4.5 mm in length; T & I Japan, Saitama, Japan) was immediately placed in the extraction socket on the right side with primary stabilization. For PL, Ti implant picked up with yellow pipets and covered bone contact area and the soft tissue contact area of it hydrophilized a using small, handheld plasma device (Piezobrush^®^ PZ2; Relyon Plazma GmbH) for 30 s with a rolling motion, and then the treated implants were immediately placed in the extraction socket on the left side with primary stabilization (Fig. 1c). The rats were housed until killing while being provided with water and solid food, and the placed implants were checked for bacterial infection, movement, and loosening until tissue sampling. All rats were euthanized under deep anesthesia at each time point: 3, 7, and 14 days after implant surgical treatment. Samples for histological evaluation (*n* = 5/time point) and total RNA extraction (*n* = 3/time point) were collected. All experiments were performed according to the Guidelines for the Treatment of Animals at Tokyo Dental College (approval No.: 213303 and 233303).

### Histological measurement of the peri-implant soft tissue sealing area

At 3, 7, and 14 days after surgery, the maxillary jaw, including the implant-placed area, was harvested (*n* = 5/time point). The tissue samples with the implant were fixed in 10% neutral buffered formalin (Wako Pure Chemical Industries, Ltd., Osaka, Japan) for 1 day and decalcified with ethylenediaminetetraacetic acid (pH 7.0–7.5, 0.5 mol/L; Wako Pure Chemical Industries, Ltd.) at room temperature for 2 weeks. The implant body was carefully removed and the specimens were embedded in paraffin according to the standard protocol. The sections were cut along the coronal plane (thickness: 3 μm) and stained with hematoxylin and eosin (H&E) for histological observation and measurement of the peri-implant soft tissue sealing area. Images of the stained sections were captured for measurement of the peri-implant soft tissue sealing area using a conventional microscope (Axiophot2; Carl Zeiss, Oberkochen, Germany).

**Fig. 2:**
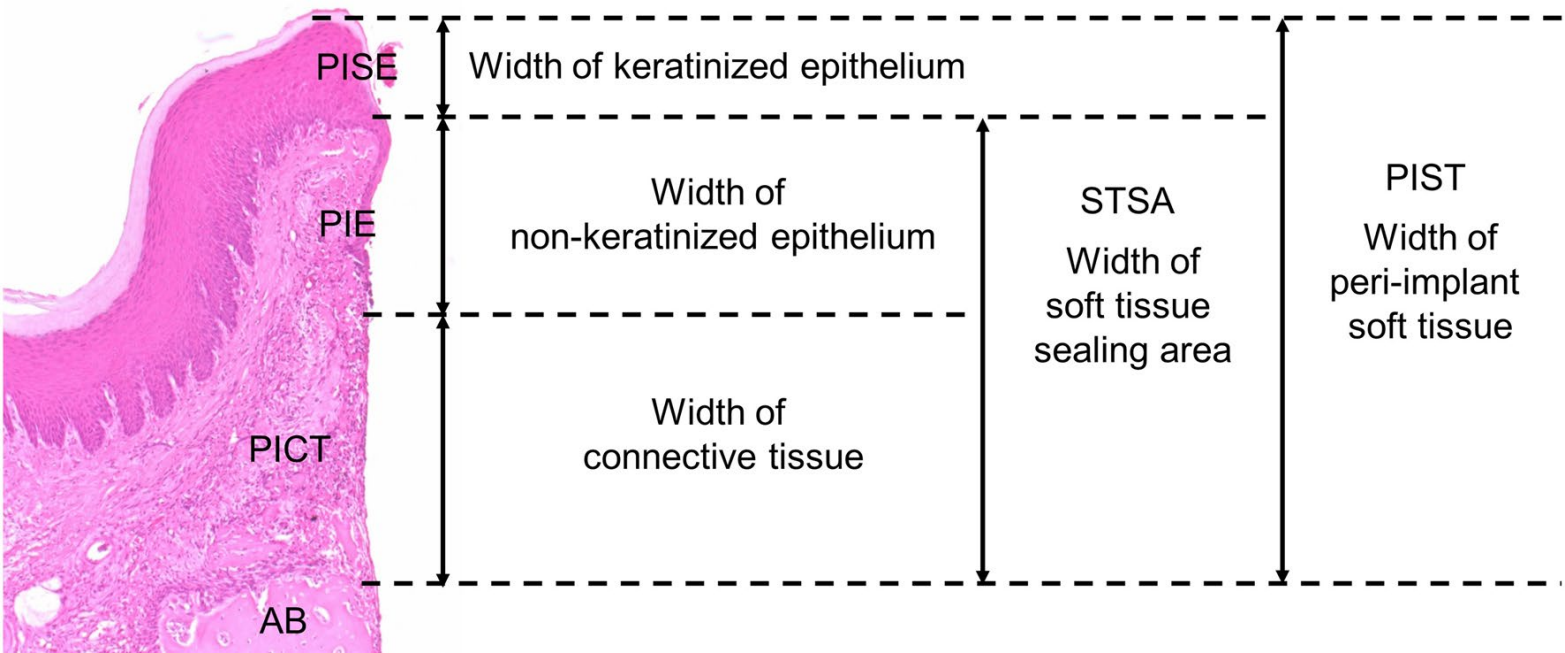
Measurement of the axial width at the peri-implant soft tissue sealed area. Representative images of H&E staining to measure the vertical width at the peri-implant soft tissue during wound healing are shown

For evaluating the peri-implant soft tissue sealing area, three sections at each time points were randomly selected from a sample (*n* = 5/time point). The axial width of the keratinized epithelium area as the peri-implant sulcus epithelium area (PISE), non-keratinized epithelium area attached to the implant as the peri-implant epithelium area (PIE), and connective tissue area attached to the implant as PICT were measured. In addition, the axial width of the soft tissue sealed area (STSA) was calculated as the total width of PIE and PICT, while that of the peri-implant soft tissue area was calculated as the total width of PISE, PIE, and PICT (Fig. 2).

### Reverse transcription quantitative polymerase chain reaction (RT-qPCR)

Tissue samples for total RNA extraction were collected using a dissecting microscope (SZ61; Olympus). All samples were washed with saline and placed in RNAlater RNA stabilization reagent (Qiagen, Valencia, CA, USA). Tissue homogenization was performed with 5 mm zirconia beads at 30 Hz for 2 min using a tissue lyser (Qiagen).

RT-qPCR was performed to evaluate collagen maturation using type 1 collagen α1 (*Col1a1*), type 1 collagen α2 (*Col1a2*), type 3 collagen α1 (*Col3a1*), and type 12 collagen α1 (*Col12a1*), and cell adhesion using integrin α2 (*Itga2*), integrin β1 (*Itgb1*), integrin α5 (*Itga5*), fibronectin 1 (*Fn1*), protein tyrosine kinase 2 (*Ptk2*), and vinculin (*Vcl*). The expression levels of target genes were determined by RT-qPCR and normalized against that of glyceraldehyde 3-phosphate dehydrogenase as an endogenous control. Total RNA was reverse-transcribed using the QuantiTect Reverse Transcription kit (Qiagen), and RT-qPCR was performed using the TaqMan Fast Universal PCR Master Mix (Thermo Fisher Scientific Inc. MA, USA) and TaqMan MGB probes (Applied Biosystems, Foster City, CA, USA) (*Col1a1*: Rn01463848_m1, *Col1a2*: Rn01526721_m1, *Col3a1*: Rn01437681_m1, *Col12a1*: Rn01521220, *Itgb1*: Rn00566727_m1, *Itga2*: Rn01489315_m1, *Itga5*: Rn01761831_m1, *Fn1*: Rn00569575_m1, *Ptk2*: Rn00433209_m1, and *Vcl*: Rn01755886_m1) in the ABI 7500 Fast Prism Sequence Detection System (Thermo Fisher Scientific Inc.). Total RNA was first incubated at 42 °C for 2 min, reverse-transcribed at 42 °C for 15 min, and then inactivated at 95 °C for 3 min. The RT-qPCR conditions were as follows: 95 °C for 20 s, 50 cycles at 95 °C for 30 s, and 60 °C for 30 s. All reactions were performed in triplicate for each sample, and the results were analyzed using the ΔΔCT method. These target gene expression patterns were compared in the Cont and PL groups during wound healing from Day 3 to Day 14.

### Picrosirius red staining for collagen fiber detection

The paraffin-embedded sections were stained with Picrosirius Red (Picrosirius Red Stain Kit; ScyTek Laboratories Inc., Logan, UT, USA) for collagen fiber detection at the peri-implant soft tissue area during wound healing. The sections were deparaffinized with xylene and ethanol and stained with Picrosirius Red Solution for 15 min. Stained sections were rinsed with 0.5% acetic acid solution and absolute ethanol. These stained sections were then examined and photographed using a conventional microscope (Axiophot 2; Carl Zeiss) for evaluation.

### Immunohistochemical evaluation for integrin α2, α5, and β1 at the biological sealing area

Immunohistochemical expression of integrin α2, integrin α5, and integrin β1 was evaluated by 3,3'-diaminobenzidine (DAB) staining. The paraffin sections deparaffinized with xylene and ethanol and washed with phosphate-buffered saline (PBS) were treated with 0.1% protease (Nichirei, Tokyo, Japan) for antigen retrieval for 3 min at 37 °C. These sections were then treated with 0.3% H_2_O_2_ in methanol at room temperature to block endogenous peroxidase and incubated with Blocking One Histo (Nacalai Tesque, Kyoto, Japan) for 30 min to block nonspecific immunoglobulin binding. For primary antibody reaction, sections were incubated with the following primary antibodies for 2 h at room temperature: anti-integrin α2 rabbit polyclonal antibody (1:200; St John’s Laboratory Ltd., London, UK), anti-integrin α5 rabbit polyclonal antibody (1:200; St John’s Laboratory Ltd.), and anti-integrin β1 rabbit polyclonal antibody (1:200; Proteintech Group Inc., Chicago, IL, USA). After washing with PBS, sections were incubated for 30 min at room temperature with secondary antibody: Histofine Simple Stain Rat MAX-PO(R) for rabbit primary antibody (Nichirei). Immunoreactions were visualized using diaminobenzidine reagent (DAB substrate kit; Nichirei), and sections were finally counterstained with hematoxylin. All sections were examined and photographed using a conventional microscope (Axiophot 2; Carl Zeiss).

### Statistical analysis

Histological measurement of the peri-implant soft tissue sealing area was performed with three sections for a sample (*n* = 5/time point). RT-qPCR was repeated three times for the two samples (*n* = 6 samples/time point). The values are expressed as means ± standard deviations. Data analysis was performed using GraphPad Prism (version 5.04; GraphPad Software Inc., San Diego, CA, USA). The levels of significance were defined as *p* < 0.01 and *p* < 0.05. Statistical analysis for the evaluation of Ti disks (wettability, roughness, and temperature change) was performed using Student’s *t*-test. Statistical analysis of histological measurements at each time point was performed using Mann–Whitney *U* test, and gene expression patterns of the Cont and PL groups during wound healing from Day 3 to Day 14 was performed using two-way analysis of variance with Bonferroni multiple comparisons.

## Results

### Effect of hydrophilization on the Ti disk by the handheld plasma device

**Fig. 3:**
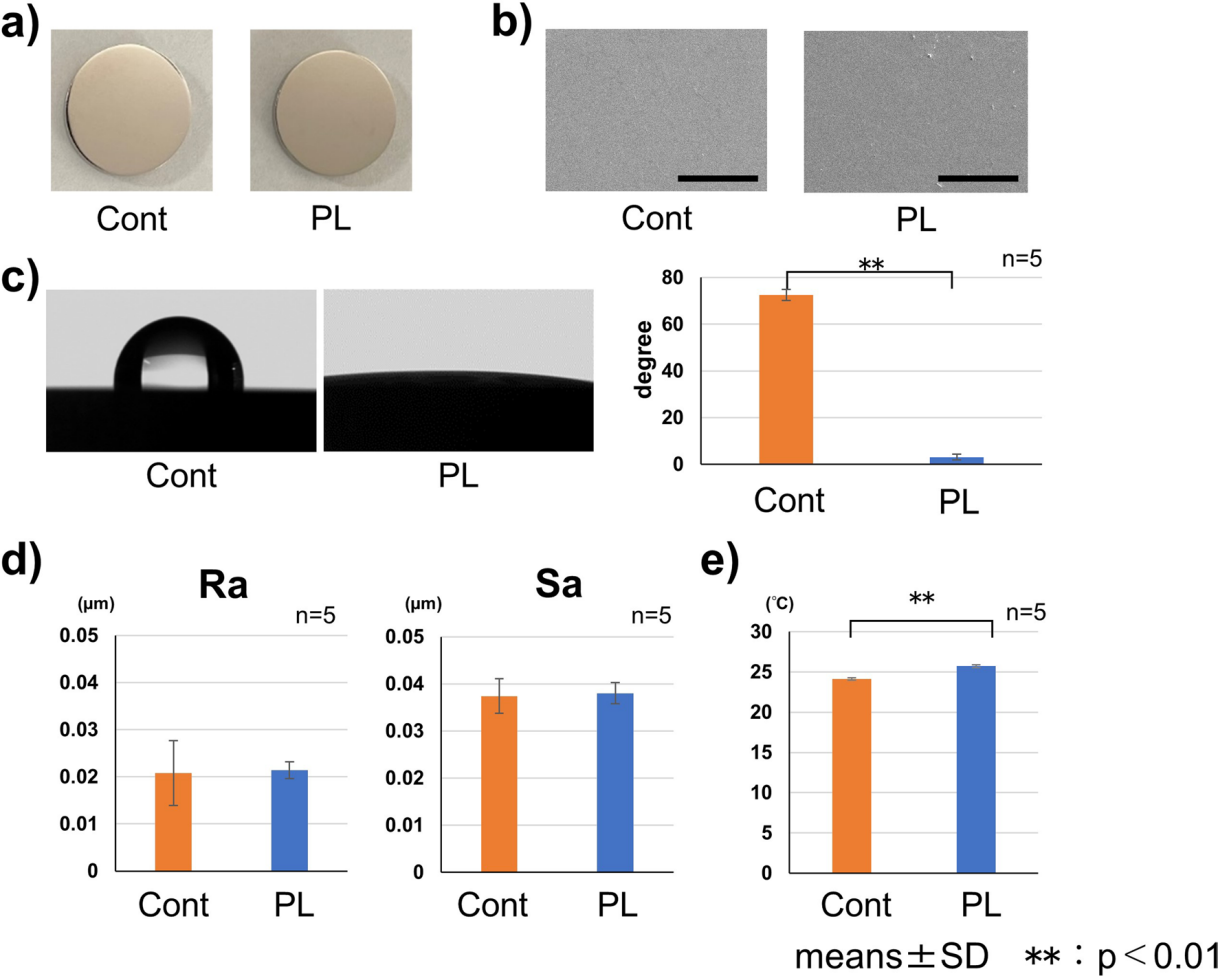
Evaluation of hydrophilization to the Ti disk. **a** Photographs, **b** SEM images (bars: 100 μm). **c** Contact angle. **d** Surface roughness (Ra: two-dimensional arithmetic mean surface roughness, Sa: three-dimensional arithmetic mean surface roughness). **e** Temperature change of the Ti disk surfaces after treatment. Values are expressed as means ± SD. **p < 0.01

The effect of plasma application to the Ti disk using the handheld plasma device was examined by surface observation, measurement of the contact angle, surface roughness, and temperature change (Fig. 3). No differences in the micro- or macro-level surface observations were found between the PL and Cont groups (Fig. 3a, b). In the contact angle measurement for hydrophilic evaluation, a low contact angle was observed on the plasma-treated disk compared with the untreated disk as a control (Fig. 3c). By contrast, no significant difference in surface roughness or temperature was seen between the PL and Cont groups (Fig. 3d, e).

### Effect of plasma application on the STSA in peri-implant soft tissue

**Fig. 4:**
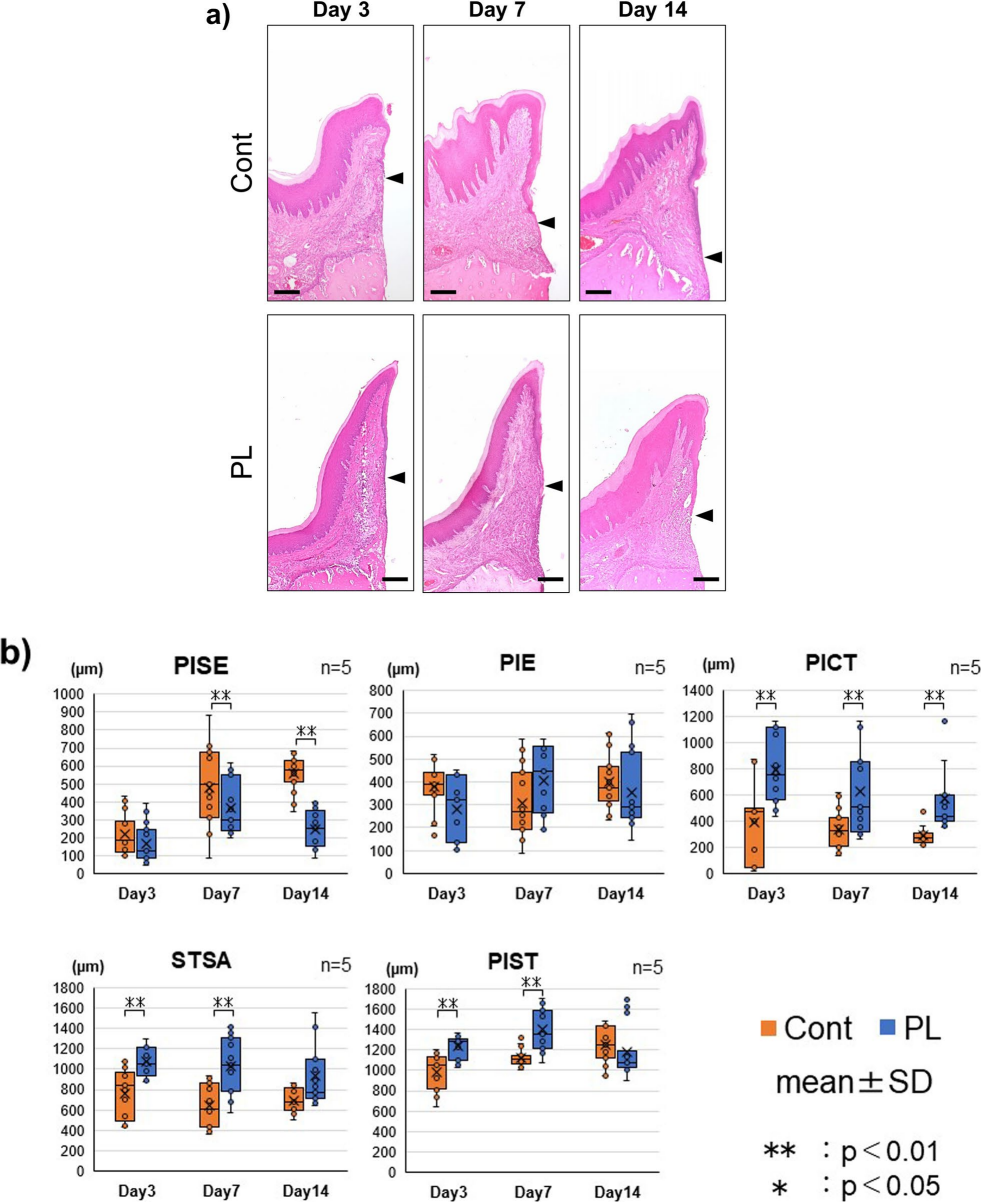
Changes in vertical width at the peri-implant soft tissue sealed area during wound healing. **a** Low magnification images of representative H&E staining for each time period used to measure each component of the peri-implant soft tissue (bars: 200 μm) are shown. The lowest points of the epithelium are indicated by arrowheads. The downgrowth of non-keratinized epithelium contacting the implant surface was recognized in both the PL and Cont groups. **b** The vertical distances of each component were measured and analyzed. Values are expressed as means ± SD. **p < 0.01, *p* < 0.05

In the histological observation of the H&E-stained sections, the downgrowth of non-keratinized epithelium contacting the implant surface was recognized in both the PL and Cont groups. In addition, no inflammation due to bacterial invasion was seen in PICT (Fig. 4a). Measurements of the soft tissue sealed areas in peri-implant soft tissue for each group are shown in Fig. 4b. The widths of the PISE (keratinized epithelium) and contactless area were significantly higher in the Cont than in the PL group at Days 7 and 14 (*p* < 0.05). No significant difference in the width of the PIE (non-keratinized epithelium) or implant-attached area was found between the Cont and PL groups. However, the width of the PICT (implant-attached connective tissue area) was significantly higher in the PL than in the Cont group at all time points (Days 3, 7, and 14; *p* < 0.01). The width of the STSA (implant contact area with peri-soft tissue) was also significantly higher in the PL than in the Cont group at all time points (*p* < 0.05). However, the PIST (total width of peri-implant soft tissue) was significantly higher in the PL than in the Cont group at Days 3 and 7 (*p* < 0.05); no significant difference was observed at Day 14.

### Effect of handheld plasma application on collagen synthesis in the peri-implant soft tissue area during wound healing

**Fig. 5:**
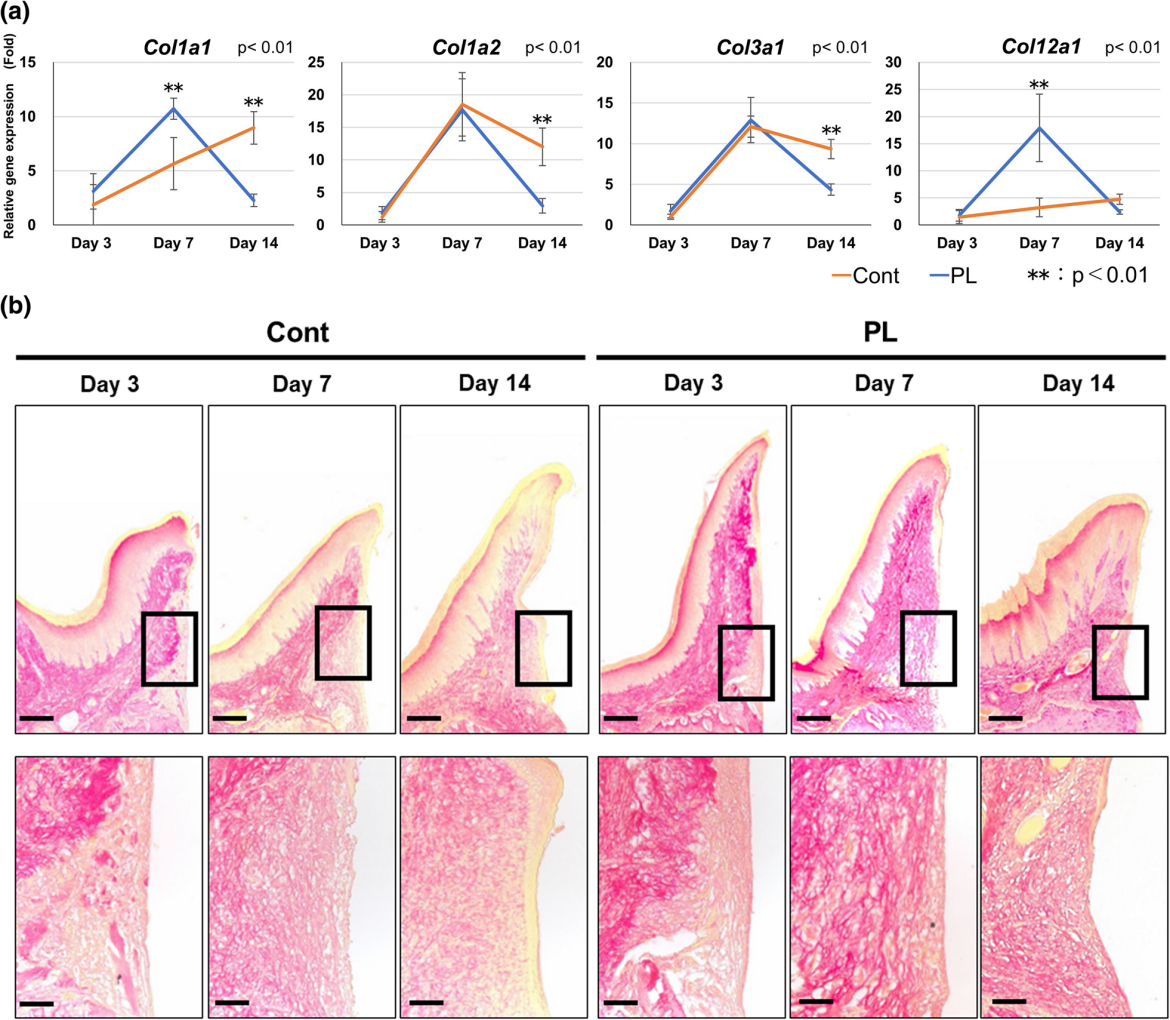
Maturation of collagen. **a** Expression of collagen maturation-related genes during wound healing. Relative gene expression levels were quantified based on Day 3 of Cont. **p < 0.01. **b** Low magnification of Picrosirius Red staining for collagen maturation during wound healing (bars: 200 μm) and medium magnification images of connective tissue around the apical part of the peri-implant epithelium are shown (bars: 50 μm). Pale stained area inside the dotted line indicates immature fibrous connective tissue. The area decreased with time

The gene expression levels of four collagen-related genes in PIST were evaluated at Days 3, 7, and 14 by RT-qPCR (Fig. 5a). The highest expression of *Col1a1* and *Col1a2* (collagen synthesis and maturation, respectively) was recognized at Day 7 in the PL group and Day 14 in the Cont group (*p* < 0.01), whereas the expression of *Col3a1* (an immature collagen marker) was reduced in the PL group but maintained in the Cont group at Day 14 (*p* < 0.01). One of the fibril-associated collagens with interrupted triple helices (FACIT), *Col12a1*, related to the cross-linking of type I collagen, was strongly expressed in the PL group at Day 7 (*p* < 0.01), but no expression was observed in the Cont group.

Localization of synthesized collagen fiber in the implant surface area of the connective tissue during wound healing was evaluated by Picrosirius Red staining (Fig. 5b). In the Cont group, a pale stained area due to immature collagen fiber was recognized at the implant-attached part of the connective tissue from Days 3 to 14, whereas the pale stained area at the implant-attached part was reduced over time in the PL group. At Day 14, a pale stained area was observed in the sub-epithelial part in the Cont group, but no immature collagen was recognized at either the implant-attached part or sub-epithelial area.

### Effect of handheld plasma application on cell adhesion-related gene expression and localization of integrin in peri-implant soft tissue

**Fig. 6:**
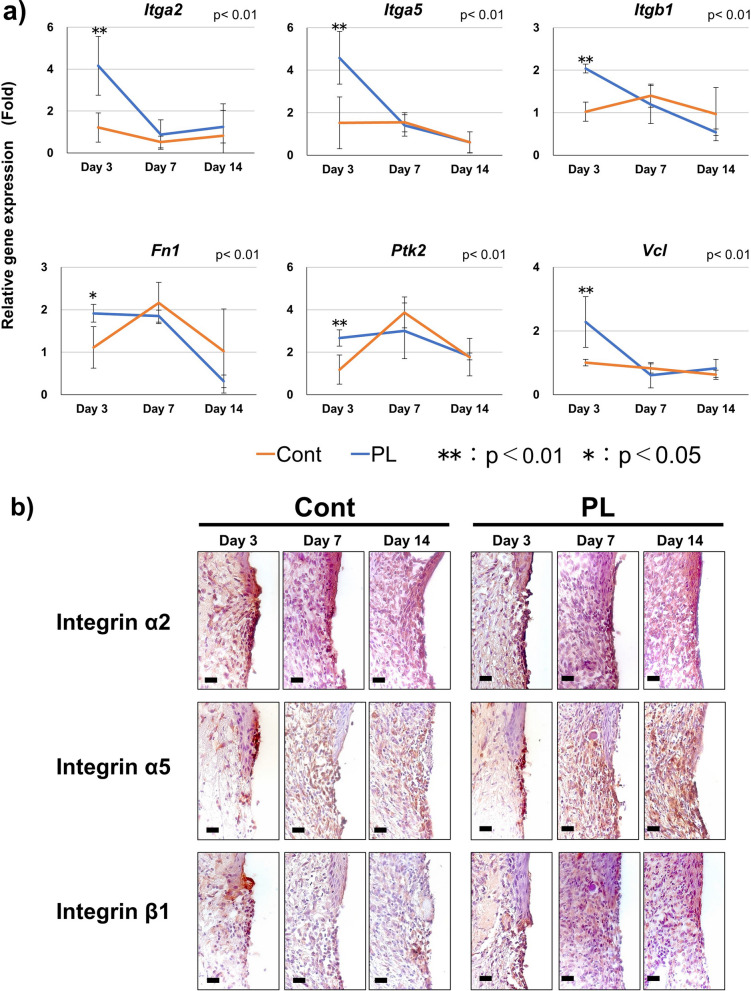
Connective tissue attachment. **a** Expression of adhesion-related genes during wound healing. Relative gene expression levels were quantified based on Day 3 of Cont. **p < 0.01, *p < 0.05. **b** Immunohistochemistry of integrin α2, α5, and β1 during wound healing of peri-implant connective tissue. High-magnification histological evaluation during wound healing in peri-implant connective tissue is shown (bars: 20 μm). The regions of interest (ROIs) for integrin localization were set at the peri-implant connective tissue around the apical part of the peri-implant epithelium (shown with H&E staining, bar: 200 μm). Immunoreaction for each integrin was observed in the peri-implant connective tissue

The gene expression levels of six cell adhesion-related genes, including integrin, in PIST were evaluated at Days 3, 7, and 14 days by RT-qPCR (Fig. 6a). Significantly higher expression of integrin α2 (*Itga2*), α5 (*Itga5*), and β1(*Itgb1*), which are known as principal transmembrane receptors involved in extracellular binding, was observed in the PL than in the Cont group at Day 3 (*p* < 0.01), but this significant difference disappeared after Day 7. The extracellular matrix binds to integrin; fibronectin (*Fn1*) and focal adhesion-associated protein; *Ptk2* and *Vcl* also highly expressed in PL group than Cont group at Day 3 (*p* < 0.05).

The localization of integrin α2, α5, and β1 in PICT during wound healing was examined by immunohistochemistry (Fig. 6b). Stronger immunoreaction of integrin α2 was observed at the implant contact area in the PL group compared with the Cont group at all time points. In addition, a positive reaction in the PL group was recognized in not only the implant contact area, but also the deep part of PICT. An immune positive reaction of integrin α5 in both the PL and Cont groups was observed in the implant contact area of connective tissue at Day 3, and a strong immune reaction in the deep part of connective tissue was recognized in the PL group from Days 7 to 14. The positive reaction of integrin β1 at Day 3 was localized strongly in the implant contact area of connective tissue adjacent to the apical part of the peri-implant epithelium in both the PL and Cont groups, and the expression was moderately maintained in the implant contact area of connective tissue at Days 7 and 14.

## Discussion

In this study, we investigated the effects of surface modification of Ti dental implants using a small, handheld, low-temperature atmospheric plasma device on wound healing in peri-implant soft tissue after implant placement. The hydrophilic effect on the Ti disk was evaluated using a wettability surface test. The results of the contact angle test showed that the application of handheld low-temperature plasma added hydrophilicity on the Ti disk. It is well known that a hydrophilized Ti surface using plasma treatment increases the adhesion of fibroblasts [34]. In a preliminary experiment, a conventional plasma device showed a temperature increase and discoloration of the Ti disk surface compared with the handheld plasma device (data not shown). We set the time for plasma application to 30 s, which seems appropriate for the chair side of clinical dental treatment. Ti shows discoloration owing to the formation of an oxide film on the surface at high temperature [35], which suggests that a handheld plasma device is a useful tool for adding hydrophilization on Ti disks without discoloration. Our results showed that the handheld plasma device had no effect on the roughness of the Ti surface. It was previously reported that the roughness of the implant abutment surface influences bacteria accumulation, and that a reduction of the Ra value to under 0.2 μm is a threshold for bacterial adhesion resistance [36]. Furthermore, low-temperature atmospheric pressure plasma treatment has been shown to inhibit bacterial adhesion [37]. These results suggest the handheld plasma device is also a useful tool for managing the peri-implant soft tissue area.

Histological observation and measurement of the peri-implant soft tissue sealing area revealed that handheld plasma application inhibits peri-implant epithelial downgrowth. Plasma application to the Ti implant increased the width of PICT and STSA compared with the untreated group and did not affect the width of PIST and PIE at 14 days after surgery. In addition, plasma application significantly increased the width of PICT at the early phase of wound healing and maintained this increased width until Day 14. These results indicate that maintaining the width of PICT using plasma application suppressed peri-implant downgrowth. In previous studies, peri-implant epithelium downgrowth was regulated by establishing peri-implant epithelial–connective tissue continuity, and implant–connective tissue attachment was a key factor in suppressing epithelial downgrowth [12, 17]. Based on these results, with a focus on the PICT area to reveal the mechanisms underlying the suppression of peri-implant epithelial downgrowth, we investigated the effect of collagen maturation and cell adhesion in PICT by handheld plasma application.

Both collagen-related gene expression and histological expression on Picrosirius Red staining indicated that collagen maturation in the peri-implant soft tissue was accelerated by handheld plasma application compared with the untreated group. The highest mRNA expression of type I collagen, which is known to be a marker of collagen synthesis and maturation, was recognized at Day 7, and was reduced at Day 14 in peri-implant soft tissue in the PL group, whereas it increased over time in the Cont group. Furthermore, the expression pattern of type XII collagen (*Col12a1*) was similar to that of type I collagen. COL12A1, which is known as one of the FACIT collagens, is present in the superficial layer of type I collagen and plays important roles in cross-linking and keeping collagen bundles together [38]. Although collagen gel contraction is generally attributed to cell–collagen interactions via integrin α2β1, type XII collagen has been reported to promote gel contraction by modulating interfibrillar interactions rather than such intracellular events [39]. The gene expression of *Col12a1* has been reported to be increased in oral fibroblasts during wound healing [40]. Therefore, the expression of FACIT collagen may result in the contraction of collagen fibers in connective tissue, and be associated with tissue sealing by the peri-implant soft tissue. On the other hand, gene expression of type III collagen in immature tissue during wound healing was higher in the Cont group than in the PL group at Day 14. The findings of the histological evaluation with Picrosirius Red staining also supported these gene expression patterns. The pale stained area due to immature collagen fiber remained in the sub-epithelial connective tissue area in the Cont group. By contrast, the pale stained area was observed as granulation tissue at the implant contact area of PICT, the same as the Cont group, but the size of the pale stained area was reduced at Day 14 in the PL group. However, the mechanism underlying the acceleration of collagen maturation in PIST by plasma application to the Ti disk remains unclear. A previous study reported that increased wettability of a Ti disk accelerates fibroblast migration in a wound healing assay [41]. In addition, direct application of wounds on the back of mice with low-temperature atmospheric pressure plasma has been shown to promote wound contraction associated with angiogenesis through the generation of reactive oxygen/nitrogen species [42]. Therefore, in the present study, cell adhesion to the implant surface by plasma application appeared to be a key factor in the early adhesion and migration of fibroblasts to the Ti surface and tissue remodeling with less immature fibrous connective tissue, thereby resulting in accelerated connective tissue maturation.

All cell adhesion-related genes, including integrins, showed high expression at Day 3 by plasma application and decreased over time. Integrins are transmembrane proteins composed of α and β subunits that link the extracellular matrix to the cytoskeleton and regulate functions such as cell adhesion, morphology, and differentiation [43]. It has been reported that fibroblasts express some integrin subunits (α2, α5, and β1) and focal adhesion-associated proteins (Ptk2 and Vcl); these integrins are known to form focal adhesions, to enhance cell adhesion to the Ti surface with high gene expression, and to be involved in the formation of tight sealing by the PICT [41, 44, 45]. The extracellular matrix is known to bind with the integrins fibronectin, vitronectin, and collagen, and the focal adhesion-associated proteins Vcl and Ptk2 have been shown to be expressed in fibroblasts at the implant surface and connective tissue interface [46]. Integrin α2β1 binds with type I collagen, which is known to be an extracellular matrix associated with cell adhesion [47] and a major component of gingival connective tissue, and to maintain the structure of the tissue [48]. By contrast, integrin α5β1 is a fibronectin receptor reported to be associated with cell adhesion and to contribute to the initial adhesion of fibroblasts [49]. Fibronectin is involved in a variety of cellular functions, including adhesion, migration, growth, and differentiation [48], is expressed on implant surfaces, and is known to increase adsorption on hydrophilic surfaces selectively [50]. In our experiment, the gene expression of type I collagen was increased and collagen maturation was accelerated by plasma application. Furthermore, the strong immunoreaction of integrin α2 was localized at the implant contact area of PICT in the experimental group. Furthermore, the gene expression of integrin α5 and fibronectin was significantly higher in the PL than in the Cont group at the early stage of wound healing. Immunohistochemical analysis of integrin α5 also showed a strong positive reaction in the PICT in the experimental groups. Hydrophilic surfaces have been reported to have an advantage in the adsorption of adhesion-related extracellular matrices and to produce more integrins, thereby promoting the maturation of focal adhesion and enhancing adhesion [41]. These results suggest that plasma application promotes fibroblast adhesion and migration on Ti as a result of the increasing adsorption of the extracellular matrix as fibronectin. In addition, this cell adhesion to the implant surface suppresses granulation tissue formation and inhibits the downgrowth of PIE during wound healing in peri-implant soft tissue.

Biological sealing in PIST is important to prevent bacterial invasion from reaching bone tissue. The suppression of epithelial downgrowth in peri-implant soft tissue can be a key factor by maintaining the vertical distance from the apical part of the epithelium to the bone tissue. Furthermore, the acceleration of cell adhesion in PICT at the early stage of wound healing also contributes to the prevention of postoperative infection for one-stage implant treatment. In a previous study, low-temperature atmospheric plasma was shown to prevent bacterial adhesion and biofilm formation on a Ti surface [37].

This study did have some limitations. First, we did not evaluate the remaining plasma effect on Ti implants after Day 14 or the prevention of bacterial infection. However, the plasma device used in this study can likely be used clinically at the chair side and be directly applied to the implant abutment and superstructure in the oral cavity because it is handheld and does not require a gas cylinder for application. In addition, plasma application does not increase the temperature compared with conventional devices. Therefore, these results could be expected to contribute to the establishment of a procedure for preventing peri-implantitis over the long term by using low-temperature atmospheric pressure plasma.

## Conclusions

In conclusion, handheld low-temperature atmospheric pressure plasma added hydrophilic properties to the Ti surface and maintained the width of the contact area of PICT to the implant surface owing to accelerated collagen maturation and fibroblast adhesion compared to no plasma application. This suggests that the application of low-temperature atmospheric plasma on titanium implants and/or abutment was important to prevent bacterial infection in peri-implant tissue by establishing biological sealing in peri-implant tissue.

## Data Availability

The data used and/or analyzed in this study are available from the corresponding author on reasonable request.

## Abbreviations

Ti: Titanium
CpTi: Commercial pure titanium
PISE: Peri-implant sulcus epithelium
PIE: Peri-implant epithelium
PICT: Peri-implant connective tissue
AB: Alveolar bone
STSA: Soft tissue sealing area
PIST: Peri-implant soft tissue
Col1a1: Type 1 collagen α1
Col1a2: Type 1 collagen α2
Col3a1: Type 3 collagen α1
Col12a1: Type 12 collagen α1
Itga2: Integrin α2
Itgb1: Integrin β1
Itga5: Integrin α5
Fn1: Fibronectin 1
Ptk2: Protein tyrosine kinase 2
Vcl: Vinculin
